# A Novel Inhibitor against the Biofilms of Non-Tuberculous Mycobacteria

**DOI:** 10.3390/pathogens13010040

**Published:** 2023-12-31

**Authors:** Parvinder Kaur, Ramya Vadageri Krishnamurthy, Radha Krishan Shandil, Rahul Mohan, Shridhar Narayanan

**Affiliations:** 1 Foundation for Neglected Disease Research (FNDR), Doddaballapur, Bengaluru 561203, Karnataka, India; ramya.vk@fndr.in (R.V.K.); rk.shandil@fndr.in (R.K.S.); shridhar.narayanan@fndr.in (S.N.); 2 National Center for Polar & Ocean Research (NCPOR), Headland Sada, Vasco da Gama 403802, Goa, India; rahulmohan@ncpor.res.in

**Keywords:** drug resistance, planktonic, biofilm, non-tubercular mycobacteria, *Mycobacterium abscessus* (Mabs), *Mycobacterium avium* (Mav), *Mycobacterium gordonae* (Mgo), *Mycobacterium kansasii* (Mkan), *Mycobacterium intracellulare* (Mint), *Mycobacterium nonchromogenicum* (Mnc), cystic fibrosis (CF)

## Abstract

Non-tuberculous Mycobacteria (NTM), previously classified as environmental microbes, have emerged as opportunistic pathogens causing pulmonary infections in immunocompromised hosts. The formation of the biofilm empowers NTM pathogens to escape from the immune response and antibiotic action, leading to treatment failures. NF1001 is a novel thiopeptide antibiotic first-in-class compound with potent activity against planktonic/replicating and biofilm forms of various NTM species. It is potent against both drug-sensitive and -resistant NTM. It has demonstrated a concentration-dependent killing of replicating and intracellularly growing NTM, and has inhibited and reduced the viability of NTM in biofilms. Combination studies using standard-of-care (SoC) drugs for NTM exhibited synergetic/additive effects, but no antagonism against both planktonic and biofilm populations of *Mycobacterium abscessus* and *Mycobacterium avium*. In summary, the activity of NF1001 alone or in combination with SoC drugs projects NF1001 as a promising candidate for the treatment of difficult-to-treat NTM pulmonary diseases (NTM-PD) and cystic fibrosis (CF) in patients.

## 1. Introduction

A NTM group of environmental mycobacteria, the erstwhile mycobacteria other than tuberculosis (MOTT) are emerging as major opportunistic pathogens [1] in immune-compromised patients with underlying lung diseases like cystic fibrosis (CF), non-CF bronchiectasis, chronic obstructive pulmonary disease (COPD) [2], renal failure, leukemia, and in organ transplant recipients [3]. NTM cause TB-like pulmonary dissemination, skin and soft tissue infections [3], lymphadenitis, and meningitis [3,4,5]. NTM pathogenesis has received relatively less attention than tuberculosis (TB) [5]. *Mycobacterium avium* (Mav) was the first NTM found to be associated with mortality in HIV patients [5,6]. This awareness led to the recognition of several disseminated NTM infections in immune-compromised patients, particularly in those with CF [4,5,6]. The last two decades have witnessed an alarming increase in pulmonary NTM infections (2/million to 98/million) [7,8,9] in CF patients [10] and in COPD patients treated with inhaled cortico-steroid therapy [11]. Currently, *Mycobacterium abscessus* (Mabs) is isolated from 3 to 10% of CF patients with poor treatment outcomes in the USA and Europe [12,13]. The situation has further worsened post-COVID-19 pandemic [14].

Incidences of NTM pulmonary infections with Mav complex (MAC; *Mycobacterium avium* and *Mycobacterium intracellulare*), Mabs complex (MABC; *Mycobacterium abscessus* subspecies *abscessus* and *Mycobacterium abscessus* subspecies *massiliense*), and other NTM species (*Mycobacterium chelonae*—Mch, *Mycobacterium kansasii*—Mkan, *Mycobacterium fortuitum*—Mfo, etc.) are rising. Mabs is particularly associated with NTM pulmonary diseases (NTM-PD) in CF patients with poor cure rates and high drug resistance [1,15]. The success of NTM can be attributed to the ubiquitous presence of multiple sps. in the environment and their ability to form biofilms that lead to phenotypic and genotypic resistance to antibiotics [15,16]. NTM have a lipid-rich hydrophobic outer membrane that ensures survival in the host or environment and on the surfaces of medical devices and implants [15,16,17,18,19] in the form of biofilm, the key driver for NTM pathogenesis. Biofilms, which are more drug-tolerant, shield NTM from host immune systems and anti-mycobacterial agents, causing treatment failures [20,21,22,23,24,25].

Though these biofilms were discovered nearly 100 years ago by Louis Pasteur [26], so far, no new NTM-specific drugs have been discovered. Additionally, NTM infections are not well differentiated clinically and co-infections with TB are a constant challenge [1]. There are no well-defined and optimized drug regimens for the treatment of most NTM infections. The clinical management of NTM-PD is based on either anti-TB drugs or other antibacterials based on the clinician’s discretion, resulting in poor clinical outcomes because NTM infections do not respond well to the classic anti-TB drug treatment [1,2,27,28].

The most common multidrug regimen recommended for MAC infections are Azithromycin (AZI)/Clarithromycin (CLA), Rifampicin (RIF)/Rifabutin (RBT), Ethambutol (EMB), and Amikacin (AMI) for a minimum period of 12 months. Usually, for MAC disease management, any of the ≥3 (active) drugs, e.g., parenteral AMI, Imipenem (IMI) or Ceftriaxone (CFX), Tigecycline (TGC), AZI/CLA, Clofazimine (CLO), or Linezolid (LIN), are options and treatment may be as long as 24 months [29]. The current drugs in the market are AMI (inhaled AMI for patients with history of treatment failure [30]), Bedaquiline (BDQ), Rifabutin (RBT), AZI (Zithromax), CLA (Biaxin), Ethambutol (EMB, Myambutol), and RIF (Rifadin, Rimactane), and their combinations are not very effective against the biofilms of all NTM [1,2,27,28]. The new combination regimens are almost serendipitous, e.g., a CLO-containing regimen has been reported to improve treatment outcomes in a chronic pulmonary Mav infection murine model [31,32,33]. The treatment of Mabs is very difficult, involving multiple injectables of fairly toxic agents such as AMI, with treatment durations of up to 24 months [34]. Other injectables are Imipenem (IMI) and Ceftriaxone (CFX), which have multiple side effects [30]. There are very few new NTM drugs in the pipeline. However, newer anti-TB drugs like BDQ and MOX are also being explored against NTM infections and NTM-PD [32,33]. Omadacycline, an aminomethylcycline and an FDA-approved drug for community-acquired bacterial pneumonia and other acute bacterial skin and skin structure infections, has been investigated for NTM. However, despite its good in vitro activity against NTM, including MABC, Omadacycline did not show much activity in the murine infection model [30]. Treatment success rates in CF patients with NTM infections are only about 45.6% [35,36].

There are no NTM-specific, systematically evaluated FDA-approved antibiotics against Mabs and MAC diseases. The drug resistance is emerging fast. A resistance of NTM to anti-TB drugs was reported soon after the first randomized controlled trial of NTM treatment by the British Thoracic Society (BTS), which found no relationship between RIF, INH, and EMB in vitro sensitivities and treatment outcomes for MAC, *M. xenopi* and *M. malmoense* [1]. Basically, there is a huge research gap in discovering new drugs against NTM infections. Hence, a concerted global effort is warranted to develop NTM-specific new drugs with differentiated mechanisms of action [7].

We report here a novel thiopeptide antibiotic NF1001 with significant activity against planktonic forms of MAC, including drug-sensitive and MDR-clinical isolates. NF1001 is potent alone by itself and in combination with the existing anti-NTM drugs. NF1001 exhibits a reduction in bacterial load in planktonic and biofilm forms of Mav and Mabs. NF1001 is synergistic with SoC drugs against the biofilms of Mav and is synergistic or additive against Mabs. Thus, NF1001 seems to be a good candidate for developing improved new therapeutic regimens for the treatment of NTM infections.

## 2. Materials and Methods

NTM strains and growth conditions: We tested *Mycobacterium abscessus* (Mabs) ATCC 19977 (*Mycobacterium abscessus* subspecies *abscessus* strain L948 [TMC 1543]), *Mycobacterium avium* (Mav) ATCC 19698, *Mycobacterium kansasii* (Mkan) ATCC 12478, *Mycobacterium nonchromogenicum* (Mnc) ATCC 19530, *Mycobacterium gordonae* (Mgo) ATCC 14470, and *Mycobacterium intracellulare* (Mint) ATCC 13950 reference strains, 31 clinical isolates of Mabs (*Mycobacterium abscessus* subspecies *abscessus*, or *massiliense*), and MAC (Mav/Mint) obtained from Dr. Charles Daley, National Jewish Hospital, Colorado, US. NTM strains were cultured in Middlebrook 7H9 broth complete medium (ADC supplemented), 0.2% Glycerol and 0.1% Tween 80, at 37 °C/5%CO_2_. The experiments with Mab, MAC (Mav/Mint), Mkan, Mnc, and Mgo were performed in the FNDR BSL-2 facility. Planktonic cultures as well as the biofilm of Mab and MAC (Mav/Mint) NTM were studied.

**Figure 1:**
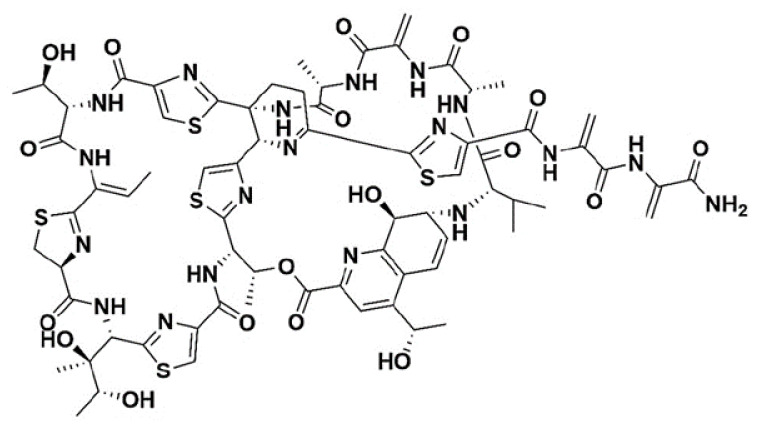
Structure of NF1001: novel, thiopeptide antibiotic. MW = 1649.49 Daltons.

NF1001: NF1001 is a natural product and a novel thiopeptide antibiotic (Figure 1) produced by *Streptomyces* sp. (*Streptomyces radiopugnans*), isolated from the Antarctica soil sample [37]. NF1001 was synthesized by fermentation by our collaborator at Anthem Biosciences Pvt. Ltd., Hosur Rd, Electronics City Phase 1, Bengaluru, Karnataka 560099, India (purity > 95%). It is highly potent against various drug (AMI/CLA)-sensitive and drug-resistant NTM species.

Reference antibiotics, drugs, chemicals, and media used: Reference antibiotics such as SoC Amikacin (AMI), Azithromycin (AZI), Rifampicin (RIF), and Clarithromycin (CLA), as well as other reference drug/s active against NTM [Moxifloxacin (MOX)] were procured from Sigma-Aldrich (St. Louis, MO, USA) (Merck, Rahway, NJ, USA) or were received as gifts from Lupin Limited (Mumbai, India). The following reagents in the category of other reference drugs [Bedaquiline (BDQ)] were obtained through the NIH, as HIV Reagent Program, Division of AIDS, NIAID, NIH: Bedaquiline Fumarate (BDQ) ARP-12702, contributed by Janssen Pharmaceuticals. Media and the supplements used in this study were Middlebrook 7H9 broth base, Middlebrook 7H10 agar base, and ADC (albumin, dextrose, and catalase); supplements were obtained from BD/Difco, Tween-80 was purchased from Merck. The stock solutions (12.8 mg/mL) of test compounds and the reference drug controls (e.g., AMI, AZI, RIF, and CLA and other anti-NTM drugs: MOX and BDQ) were prepared separately in dimethyl sulfoxide (DMSO). The compound is soluble in DMSO upto 512 mg/mL concentrations. Working solutions were freshly prepared at the time of the experiment.

Minimum inhibitory concentration (MIC) of NF1001 against the planktonic form of NTM strains: The NTM strains (Mav, Mgo, Mabs, Mkan, Mint, Mnc) were grown in the replication/planktonic phase. MIC of NF1001 was performed against the planktonic population of NTM with suitable modifications, as recommended by the Clinical and Laboratory Standards Institute guidelines M24 [38,39], by using 7H9 media, as previously described for Mtb [40]. Briefly, the test compounds were dissolved in DMSO and serially double-diluted in a 10-concentration dose response (10c-DR) ranging from 64 to 0.125 μg/mL in 96-well plates. Middlebrook 7H9 broth (supplemented with 10% ADC) complete medium was used for the assay. NTM cultures were added as 200 μL total assay volume in each well to all columns except the medium control (200 μL medium was added) column to give a final inoculum of 3–7 × 10^5^ cfu/mL. The QC included media controls, growth controls, and the reference antibiotics (AMI, AZI, RIF, MOX, BDQ, CLA). The growth controls included the respective solvent (e.g., DMSO) at the final concentration of 2% in culture (as in test wells) to maintain the identical growth conditions in the drug-containing vs. no drug-containing wells. The assay plates were incubated at 30 °C (Mabs) or 37 °C (Mav), and the results were noted on the 4th day for Mabs, Msm, and Mgo, and 6th day for Mav, Mint, Mkan, and Mnc using a turbidometric readout. The clear wells indicated inhibition of growth, while the turbid wells indicated uninhibited growth. MIC was the minimum concentration of molecules that completely inhibited the turbidometric growth of bacteria. MIC assays were carried out in duplicate (biological replicates).

Determination of minimum bactericidal concentration (MBC): MBC was determined by a procedure reported previously by us [39] from a parallel set of MIC plates. Each dilution of the inhibited culture wells from the MIC plate (25 µL) was plated with serial dilutions in triplicate (technical replicates) onto Middlebrook 7H9 agar supplemented with 10% OADC and incubated at 37 °C, respectively, for different NTM strains. The CFU was enumerated after the respective incubation period. MBC was recorded as the lowest concentration that killed 99% of the initial NTM inoculum.

Going forward, only Mabs and Mav, the two medically important NTM, were taken up for further studies.

Killing kinetics of NF1001 against NTM strains: Killing kinetics assay on planktonic population of NTM strains was performed as described earlier [39,40,41,42] for Mtb with slight modifications. The respective NTM cultures were inoculated at ~3–8 × 10^7^cfu/mL inoculum in fresh Middlebrook 7H9 complete medium, containing varying concentrations of the compound NF1001 (0.015–32 µg/mL) and were incubated at appropriate temperatures for different time points. For the CFU enumeration, aliquots from the cultures containing different concentrations of the compounds were collected at different time points for NTM (Mabs—3 days, and Mav—7 days) and plated at various dilutions (10^−1^ to 10^−8^) to obtain countable colonies. MOX was used as quality control for the assay. The data from the biological triplicate set was analyzed and plotted as log_10_ cfu/mL as a function of the concentration of NF1001 to calculate the range of the concentration showing killing potential. Emax values were calculated as the maximum kill was observed from the starting cell number at the maximum concentration/s tested in the assay.

Cytotoxicity against mammalian cell lines: Cytotoxicity of NF1001 was evaluated against different cell lines: HepG2, A549, and PMA-activated THP-1 macrophage cell lines [42]. Briefly, HepG2 (ATCC HB-8065), A549, or THP-1 monocytes (ATCC TIB-202) were maintained in the RPMI 1640 medium supplemented with 2 mM L-glutamine and 10% heat-inactivated fetal bovine serum (FBS) at 37 °C in a humidified atmosphere of 5% CO_2_. FBS was obtained from Life Technologies. Resazurin and trypan blue were purchased from Sigma-Aldrich. Further, THP-1 cells in RPMI were activated using 50 nM of phorbol 12-myristate 13-acetate for 48–72 h at 37 °C/5% CO_2_. HepG2 and A549 cells were grown as a monolayer. Post maturation of THP-1 cells into macrophages, the THP-1 macrophages, along with HepG2 and A549 cells, were exposed to test compound NF1001 (2-fold dilutions 64–0.025 µg/mL) in duplicate, incubated at 37 °C/5% CO_2_ for 48 h. Post incubation, resazurin dye was added at 0.125 mg/mL concentration with an equal volume of RPMI medium and was further incubated for 24 h. The colorimetric readings were taken 24 h after the addition of resazurin dye [42].

Intracellular Efficacy (IE) of NF1001 against NTM in THP-1 macrophages: To test drug efficacy against slow or non-replicating bacilli in the intracellular compartment, a tumor macrophage-derived cell line THP-1 was used. The THP-1 cells were grown in RPMI medium (Gibco-BRL Life Technologies, Gaithersburg, MD, USA) and supplemented with 1 mM sodium pyruvate, 2 mM L-glutamine, 1500 mg of sodium bicarbonate (Sigma, St. Louis, MO, USA) per liter, and 10% fetal calf serum (Gibco-BRL Life Technologies) without any antibiotics. The viable cells were seeded in 96-well plates (Nunc, Roskilde, Denmark) with complete RPMI at a density of approximately 5 × 10^5^ cells/well and incubated overnight. The THP-1 cells were activated by 50 nM phorbol 12-myristate 13-acetate (PMA) to achieve macrophage-differentiated phenotypes and incubated at 37 °C/48–72 h/5% CO_2_ atmosphere. THP-1 macrophages were infected with NTM strains at a multiplicity of infection (MOI) of 1:10 [41,42] and were incubated for 2 h at 37 °C with 5% CO_2_. After washing twice with phosphate-buffered saline (Ca^2+^, Mg^2+^) to remove the free bacteria, they were treated with Amikacin (50 µg/mL final concentration) at 37 °C for 2 h to kill the extracellular mycobacteria, if any were remaining. After PBS wash, the wells were replenished with fresh complete RPMI. The monolayers in the wells were washed to remove the extracellular bacteria if any were released before lysis of macrophages. Cells were lysed (0.05% SDS) at specific time points and CFU was enumerated to estimate the numbers of intracellular NTM 2 h post-infection. The lysate was enumerated. The remaining wells were used for infection controls, or for the drug-exposure at 2 h post-infection. After treatment with NF1001 (32–2 µg/mL) as well as the control drugs RIF, MOX, and CLA (at 32 to 0.5 µg/mL), the cultures were sampled from the infection control, test compound, and the reference drug control wells with each drug concentrations on D-0 (infection control for both Mabs and Mav), D-3 for Mabs, and D-7 for Mav. The cell lysates were enumerated on 7H10 agar plates for residual intracellular NTM. The killing curves were generated by plotting the log_10_ cfu/mL against different drug concentrations (µg/mL). The assay was performed as biological triplicates.

Combination MIC studies of NF1001 with SoC-NTM drugs: In vitro combination MIC studies were performed as described previously [40]. Briefly, the combination studies to decipher synergistic/additive/antagonist interactions of NF1001 with INH, RIF, AMK, CLA, and MOX were tested against 2 NTM strains (Mabs, and Mav) in 96-well plates by checkerboard method. Each combination was prepared in such a way that the middle concentration of each molecule equaled its MIC. Serial dilutions were prepared in subsequent wells. A 200 μL total volume of respective NTM cultures was added at an inoculum of about 3–8 × 10^5^ CFU/mL in each well. The culture plates were incubated at 37 °C/4–6 days as per the growth conditions. The results of inhibition were noted by turbidometry. MIC values of each drug alone and in combination were described as the lowest concentrations showing no visible growth (of respective NTM). Combinatorial reductions in MICs were used to calculate the fractional inhibitory concentration (FIC). FIC indices (FICI) were interpreted as follows: ≤0.5, synergism; >0.5–4.0, additive or indifference; and >4.0, antagonism.

MIC of NF1001 against clinical isolates of NTM strains: MIC of NF1001 was tested against a total of 31 clinical isolates of Mabs and MAC (Mav and Mint) in parallel to the respective 3 WT reference (ATCC) strains (one each for Mabs, Mav and Mint), as per the method described in the WT MIC section. NTM isolates represented a distribution of drug sensitivity (AMI-S, CLA-S), mono-resistance (AMI-R, CLA-R), and MDR (AMI-R + CLA-R) status across the strains. Mabs included WT drug sensitivity (*n* = 1), clinical isolates: drug sensitivity (*n* = 3), AMI-R (*n* = 5), CLA-R (*n* = 7), and MDR (*n* = 4). MAC (Mav/Mint) strains’ distribution included Mav: WT drug sensitivity (*n* = 1), clinical drug sensitivity (*n* = 2), AMI-R (*n* = 2), CLA-R (*n* = 2), and MDR (*n* = 1), and for Mint the strains were distributed as: WT drug (AMI/CLA) sensitivity (*n* = 1), clinical AMI/CLA sensitivity (*n* = 1), AMI-R (*n* = 3), and MDR (*n* = 1).

Biofilms of Mabs and Mav: The activity of NF1001 was evaluated against the Biofilm phase of Mabs (ATCC 19977) and Mav (ATCC 19698).

NTM Biofilms in 12-well format: The NTM cultures Mabs ATCC 19977 and Mav ATCC 19698 were grown for biofilm formation in Sauton’s media (+10% ADC) at 37 °C with shaking at 50 rpm. Mabs was grown for 1 week, and Mav for 2 weeks, in Sauton’s media with 10% ADC [43]. The cultures were diluted to 3–7 × 10^6^ cfu/mL in the respective media and were dispensed into 12-well plates (4.5 mL/well). The 12-well plates containing the respective cultures were tightly wrapped in Parafilm so that the culture plates were airtight.

NTM Biofilms in 96-well format: Though biofilms in 12-well plates were well formed, they posed limitations for running high throughput screening and the combination MICs that needs a greater number of test wells. Therefore, we developed biofilms of Mabs and Mav in 96-well format to increase the possibility of high throughput screening against NTM biofilms. The combination MIC was performed in a 96-well biofilm format. The cfu reduction in biofilm by NF1001 and SoC drugs was comparable between the 12-well and the 96-well plates. The activity of NF1001 was checked in Mabs and Mav biofilms. There are several methods available in the literature for making biofilms of different microbes, the most common being the Calgary Biofilm Device (CBD) with its pegs projecting in 96-well format, around which the biofilm forms. But, the disadvantage, particularly for the mycobacteria, is that the biofilm does not remain intact once the plate is opened. Hence, we used the mycobacterial biofilm method of Kulka et.al. [43] to test the potential of NF1001 against a stable and visibly strong biofilm. This method has been well established for mycobacterial biofilms, including Mtb [43]. Before testing NF1001 against the biofilms in 96-well format, we validated our assay using untreated as well as treatment potentials of the reference SoC drugs (AMI, AZI, and MOX) against biofilm as biological triplicates to understand the robustness and power of the assay.

Treatment of preformed biofilm using reference drug treatment: The well-formed biofilms of Mabs and Mav were tested for inhibition of intact biofilm and cfu reduction by using three different reference drugs, AMI, AZI, and MOX, at three doses (256, 64 and 16 µg/mL for all the drugs; except for MOX against Mabs, which were 64, 16 and 4 µg/mL). The validation study was performed in triplicate (biological replicates) to determine the power of the assay using an appropriate statistical analysis tool. The Mabs and Mav biofilms were exposed to the compounds diluted as 3-concentration-DR (4-fold). Appropriate no-drug control was used as the growth control (non-drug treated). The plates were wrapped again in Parafilm to maintain the airtight condition as described earlier. The sets were incubated at 37 °C, in static conditions (Mabs for 1 week and Mav for 2 weeks). At the end of the incubation time, Tween-80 (0.1%) was added to the wells and the plates were incubated at RT for 15 min. The biofilm and the entire contents were transferred to 1.5 mL Eppendorf tubes and were pelleted by centrifugation (10,000 rpm/20 min). The cell pellet was resuspended in fresh medium, and the residual cfu was enumerated on 7H10 agar plates. MIC was taken as the first concentration showing inhibition as the cfu reduction by ≥0.2 log_10_ dropped in the drug-treated samples compared to the growth control (untreated).

Exposure of preformed biofilms to test compound NF1001: After completing validation studies with robust data, the activity of test compound NF1001 was tested against Mabs and Mav biofilms. The compounds were diluted as 4-concentration-DR (4-fold), along with one no-drug control taken as the untreated/growth control. In parallel, the same assay was set up with planktonic cells as well. After observing minimal variations from biological triplicates in the validation experimental set and discerning the power of each replicate, this set of final assays using test compounds like NF1001 was further performed in triplicates (biological replicates). The MIC of NF1001 and reference QC drugs performed against biofilm and planktonic forms were plotted as net residual cfu (log_10_ cfu/mL) for comparison.

Combination MIC/activity of NF1001 along with five SoC drugs (AMI, AZI, RIF, MOX, BDQ) against NTM biofilms. In vitro drug combination MIC studies of NF1001 were performed with SoC (RIF, MOX, AZI, BDQ, and AMI) against Mabs ATCC 19977 and Mav ATCC 19698 to evaluate synergistic/additive/antagonist interactions. The compound dilutions were prepared in 96-well plates by checkerboard method as described previously [39,44]. The compounds were added carefully to the mature biofilms as described in the biofilm validation section. The compound-exposed biofilm plates were packed, wrapped in Parafilm, and incubated at 37 °C in static conditions (Mabs for 1 week and Mav for 2 weeks). At the end of incubation time, Tween-80 (0.1%) was added to the wells and the plates were incubated at RT/15 min. These were processed for residual cfu enumeration on 7H10 agar plates as described earlier and in the biofilm section [39].

Activity/MIC of NF1001 and the SoC drugs (alone and in combination) against biofilms of Mabs ATCC 19977 and Mav ATCC 19698 was evaluated by cfu/mL reduction, analyzed, and the graphs were plotted in GraphPad prism V9. The combinatorial reductions in MIC values were used to calculate the fractional inhibitory concentration (FIC). Fractional inhibitory concentration indices (FICI) were interpreted as follows: ≤0.5, synergism; >0.5–4.0, addition or indifference; and ≥ 4.0, antagonism [39,44].

Statistical analysis: The experiments performed as biological replicates are demonstrated as mean ± standard deviation (SD) with one-way ANOVA in GraphPad Prism (GraphPad Software version 9.00, La Jolla, CA, USA). The statistically significant values were demonstrated at a 95% confidence level with a **** *p* value (less than 0.0001) using Dunnett’s multiple comparisons test.

## 3. Results

The MIC and MBC of NF1001 against the planktonic form of NTM strains: NF1001 has an MIC ranging from 0.06 to 2 µg/mL against various strains of NTM in the planktonic phase of growth (Table 1). The compound demonstrated potent bactericidal activity against all the tested NTM strains (MBC values ranging from 1 to 4 µg/mL).

Table 1 shows an overview of the pan-NTM activity of NF1001 across different species.

**Figure 2:**
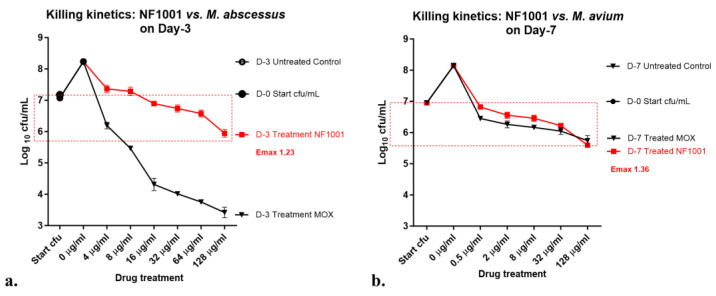
NF1001-Killing kinetics at various drug concentrations against Mabs and Mav: Symbols (residual log_10_ cfu/mL) are uniform across the graphs of respective cultures (Mabs, Mav) in 7H9 + 10% ADC: Filled circles = Day-0 start cfu- control culture unexposed to drug, Open circles = end-point control culture unexposed to drug (Day-3 for Mabs and D-7 for Mav), filled square in red = exposure to NF1001, filled inverted triangles = exposure to MOX. Data represent the mean ± SD of three biological replicates. Emax (log_10_ cfu reduction/mL at maximum concentration tested) upon NF1001 exposure vs. D-0 control culture: (**a**) Mabs ATCC 19977 (30 °C, for 3 days) Emax_128_ = 1.2, and (**b**) Mav ATCC 19698 (30 °C, for 7 days) Emax_128_ = 1.36. The red dotted box shows the quantum of maximum cfu reduction for the respective drugs.

The killing kinetics of NF1001 against NTM strains. NF1001 is bactericidal against the replicating populations of both the NTM strains: Mabs and Mav (Figure 2). An effect in the form of Emax was calculated as the maximum kill (log_10_ cfu/mL) by that particular drug at the maximum concentration tested. The Emax (log_10_ cfu/mL) of NF1001 at 128 µg/mL was in the order of Mav ATCC 19698 (1.36) > Mabs ATCC 19977 (1.23). NF1001 demonstrated an overall equally good Emax at the maximum concentration of 128 µg/mL (Emax_128µg/mL_) against both the strains as an action of increasing concentration of compounds. MOX was used as a positive control in the assay. MOX was relatively much more potent than NF1001 against Mabs. The Emax of MOX was calculated at 128 µg/mL, the maximum concentration tested. The Emax_128µg/mL_ of MOX vs. NF1001 at equivalent concentrations of 128 µg/mL was as follows: Mabs (3.75 vs. 1.23) and Mav (1.36 vs. 1.16).

The cytotoxicity against mammalian cells: NF1001 did not demonstrate any toxicity to THP-1, HepG2, and A549 cells even up to 128X MIC (cytotoxicity > 64 µg/mL), the maximum concentration evaluated. MOX, AMI, and RIF were used as the controls. All of these drugs were non-cytotoxic up to 64 µg/mL (Table 2). The cytotoxicity-positive control drug menadione showed cytotoxicity between 4 to 8 µg/mL.

**Figure 3:**
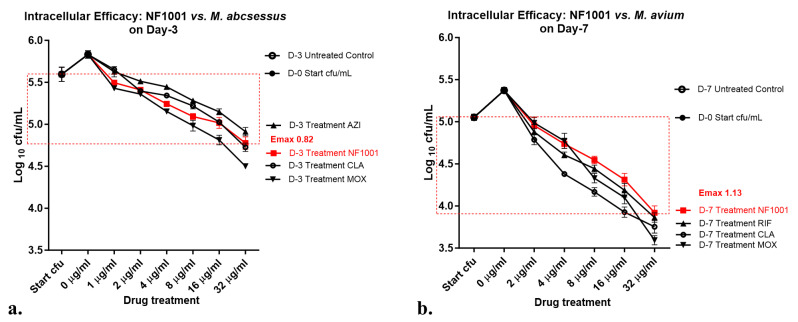
NF1001’s Intracellular efficacy (IE) at various concentrations against Mabs and Mav in THP-1 macrophages: IE was determined for NF1001 along with the reference drugs (MOX, CLA, RIF, or AZI) as biological triplicates from 32 ug/mL (two-fold five or six concentrations DR vs. no drug control) in THP-1 macrophages infected with either Mabs or Mav. Symbols (residual log_10_ cfu/mL after 3 day for Mabs and 7 days for Mav) are uniform across the graphs of Mabs and Mav: Filled circles= Day-0 start cfu- control culture unexposed to drug, Open circles = end-point control culture unexposed to drug (Day-3 for Mabs and D-7 for Mav), filled square in red = exposure to NF1001, filled inverted triangles = MOX, filled upright triangles = AZI (Mabs) and RIF (Mav), and open circles = exposure to CLA. Data represent the mean ± SD of three biological replicates. Emax (log_10_ cfu reduction /mL at maximum concentration tested) for NF1001 compared to the D-0 control culture: (**a**). Mabs ATCC 19977 Emax_32_ = 0.82, and (**b**). Mav ATCC 19698 Emax_32_ = 1.13. Red dashed box shows the magnitude of cfu drop.

The Intracellular Efficacy (IE) of NF1001 against NTM in THP-1 macrophages. NF1001 exhibited a good dose-dependent intracellular efficacy (IE) against the different NTM strains tested. The IE Emax_32µg/mL_ (log_10_ cfu/mL reduction at 32 µg/mL) against two different NTM ranged from 0.82 to 1.13 log_10_ cfu/mL. NF1001 demonstrated a 1.13 log_10_ cfu/mL reduction of Mav, while the maximum kill observed against Mabs was only an 0.82 log_10_ cfu/mL reduction (Emax = 0.82) (Figure 3).

Overall, NF1001 demonstrated activity against all the NTM species tested with an MIC in the range of 0.06 to 1 µg/mL. NF1001 was found to be active against both Mabs and Mav, but more active against MAC (Mav and Mint) compared to Mabs. NF1001 was comparatively less active against Mabs in macrophages, showing a bacteriostatic effect. Intracellular Mabs were relatively difficult to be killed versus other NTM when tested under in vitro conditions. Among the reference drugs, MOX and CLA demonstrated a better profile than RIF. NF1001 demonstrated a good in vitro and ex vivo potency profile very close to the SoC drugs.

**Figure 4:**
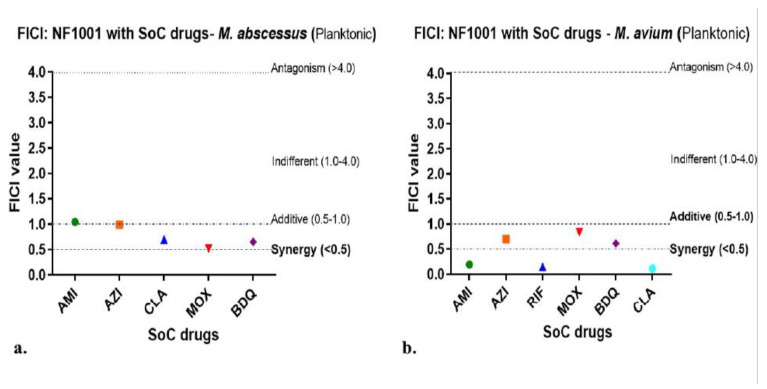
In vitro combination studies of NF1001 with various SoC drugs against planktonic NTM. (**a**). AMI, AZI, MOX, BDQ, and CLA against Mabs ATCC 19977. (**b**). AMI, AZI, RIF, MOX, BDQ, and CLA against Mav ATCC 19698. The FICI values were plotted for Mabs (**a**) and Mav (**b**) as these values appear in different zones of drug-drug interaction outcomes, e.g., synergy, additive, indifferent or antagonism. The symbols are: Filled circles in green = AMI, filled squares in orange = AZI, filled upright triangles in blue = CLA (Mabs) and RIF (Mav), filled inverted triangles in red = MOX, filled diamonds in purple = BDQ. Fractional inhibitory concentration (FIC) indices (FICI) were interpreted as: ≤0.5, synergism; >0.5–4.0, additive or indifference; and >4.0, antagonism. FICI values for Mabs (**a**) and Mav (**b**), respectively, in the range of 0.5–1.0 and 0.11–0.83. No antagonism was observed (FICI < 4.0), indicating no interaction.

The combination MIC studies of NF1001 with SoC-NTM drugs: NF1001 did not show any antagonism with any of the SoC drugs against both the NTM tested (Mabs, Mav). NF1001 was either synergistic or additive. NF1001 demonstrated additive behavior in combination with AMI, CLA, MOX, and AZI against Mabs. On the other hand, NF1001 in combination with AMI, CLA and RIF showed synergy against Mav (Figure 4).

The MIC of NF1001 against clinical isolates of NTM strains: NF1001 demonstrated pan-NTM activity against clinical isolates irrespective of their drug resistance profiles. It inhibits all the clinical isolates across different species of NTM, including drug-sensitive (AMI-S, CLA-S) and MDR (AMI-R, CLA-R, AMI-R + CLA-R) clinical isolates tested with a potent MIC range (0.06–2 µg/mL). The isolates included 20 Mabs, 8 Mav and 6 Mint isolates belonging to different antibiograms such as those that are drug-sensitive (SEN: AMI-S, CLA-S), AMI-resistant (AMI-R), and CLA-resistant (CLA-R), as well as the MDR (AMI-R + CLA-R) strains of NTMs (Table 3). NF1001 inhibited all the clinical isolates of NTM, including drug-sensitive (AMI-S, CLA-S) and MDR isolates across three species with improved MIC (range 0.06–2 µg/mL) values. Among Mabs strains, the MIC range was 0.125–1 µg/mL across 20 different drug-sensitive/resistant isolates of Mabs such as AMI-SEN/CLA-SEN (*n* = 1 WT, +3 clinical), AMI-R (*n* = 5), CLA-R (*n* = 7), and the MDR (*n* = 4: AMI-R + CLA-R). NF1001 demonstrated very good activity against MAC (Mav and Mint) strains as well with a MIC range of 0.25–2 µg/mL across the eight Mav drug-sensitive or -resistant isolates, i.e., AMI-SEN/CLA-SEN (1 WT, +2 clinical), AMI-R (*n* = 2), CLA-R (*n* = 2), and the MDR (*n* = 1, AMI-R + CLA-R), and across the six Mint strains with a MIC range of 0.06–1 µg/mL such as AMI-/CLA-SEN (1 WT, +1 clinical), AMI-R (*n* = 3), and the MDR (*n* = 1, AMI-R + CLA-R).

The MIC range (µg/mL) was deduced from the triplicate data set. The shaded boxes represent drug-R strains. NF1001 showed potency (0.06–2 µg/mL) across thirty-four different NTM:

Total 34 NTM: ten AMI-R (Mabs = five, MAC = five), nine CLA-R (Mabs = seven, Mav = two), six CLA-R + AMI-R (Mabs = four, MAC = two), and nine AMI-S/CLA-S NTM (Mabs = four, MAC = five).

Mabs (MIC range 0.125–1 µg/mL) showed a total 20 drug-S/drug-R isolates [AMI-SEN/CLA-SEN (*n* = 4); AMI-R (*n* = 5); CLA-R (*n* = 7); MDR (*n* = 4: AMI-R + CLA-R)].

MAC (Mav + Mint) (MIC range 0.06–2 µg/mL) showed a total of 14 drug-S/drug-R isolates (AMI-SEN/CLA-SEN (*n* = 5), AMI-R (*n* = 5), CLA-R (*n* = 2), MDR (*n* = 2: AMI-R + CLA-R)]

The development of NTM Biofilms in the 12-well and 96-well formats: Physically intact and strong biofilms were formed in both 12-well and 96-well plates (Supplement Figure S1).

**Figure 5:**
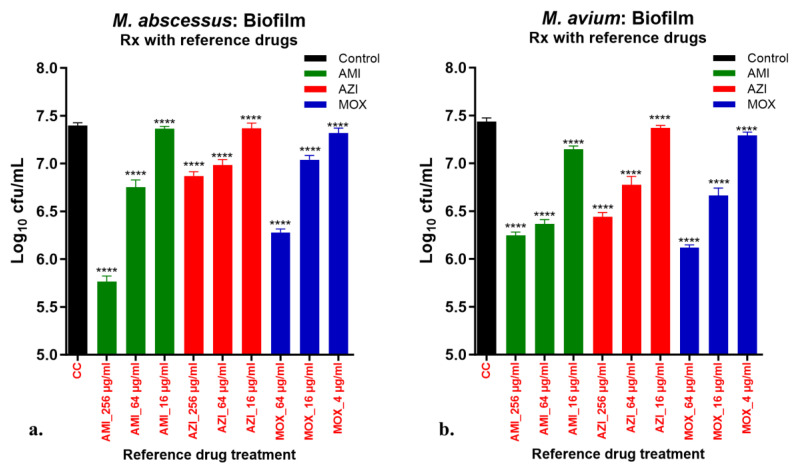
Treatment of preformed biofilm inhibition and cfu reduction using reference drug treatment: Biofilms of Mabs (**a**) and Mav (**b**) in 96-well format were tested in triplicate (biological replicates) for inhibition and cfu reduction using AMI, AZI, and MOX at three doses (256, 64 and 16 µg/mL for all the drugs; except for MOX against Mabs which were 64, 16 and 4 µg/mL) at 37 °C, along with an untreated control. The graphs represent residual log_10_ cfu/mL of Mabs (**a**) or Mav (**b**), respectively. (MIC is the first concentration showing growth inhibition or cfu reduction >0.2 log_10_ in the drug treated samples vs. the growth control.) Growth control = black bars, AMI = green bars, AZI = red bars, and MOX = blue bars. Error bars indicate ± SD of biological triplicates. Statistical significance was evaluated by one-way ANOVA (Dunnett’s test) compared to the untreated (bacteria only) control (**** *p* < 0.0001).

The validation of Mabs and Mav biofilms for disruption and cfu reduction by reference drugs: The biofilms of Mabs and Mav were susceptible to disruption by the reference drugs and exhibited modest cfu reductions (Figure 5). It is evident that there was a depletion of nutrients in the biofilm phase, hence the bacterial population reached the stationary state. AMI and MOX demonstrated the best response (>1log_10_ cfu reduction), followed by AZI (<1log_10_ cfu reduction), against Mabs or Mav biofilms. The 96-well format of biofilms was very user-friendly and easy to process through the various steps, which resulted in very robust data from independent biological triplicates (mean ± standard deviation in the range of 0.03–0.06). The power of the assay for treatment versus the untreated control groups was strong, robust, and statistically significant (*p* value **** < 0.0001) by one-way ANOVA using Dunnett’s multiple comparisons test. The ability of the 96-well assay format from validation studies encouraged us to use it for screening our test compounds. The screening of NF1001 along with the reference drugs controls was performed against Mabs and Mav biofilms for MIC and combination MIC assays.

**Figure 6:**
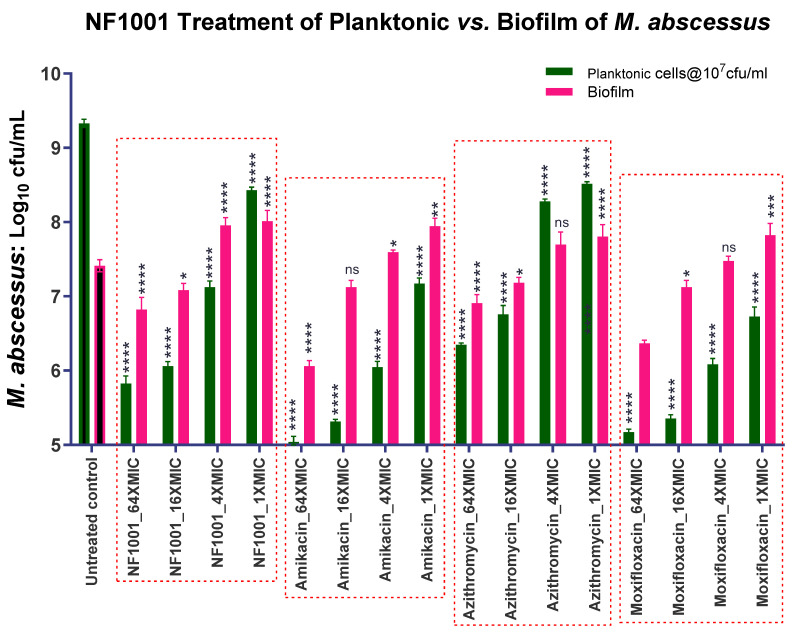
MIC/activity of NF1001 and SoC drugs against Mabs biofilm. Matured Mabs biofilms exposed to compound/drugs for three days at 37 °C. Data represent the mean ± SD of three biological replicates. Statistical significance was evaluated by one-way ANOVA (Dunnett’s test) compared to the untreated (bacteria only) control (**** *p* < 0.0001, *** *p* < 0.001, ** *p* < 0.01, * *p* < 0.1, or, ns = non-significant). The graph represents residual log_10_ cfu/mL of Mabs. Green filled bars represent the log_10_ cfu/mL of planktonic populations, while the filled pink bars show the net log_10_ cfu/mL of biofilm populations compared to the respective untreated controls. The untreated control bars represent the respective growth/cfu increase in the planktonic and the biofilm populations.

**Figure 7:**
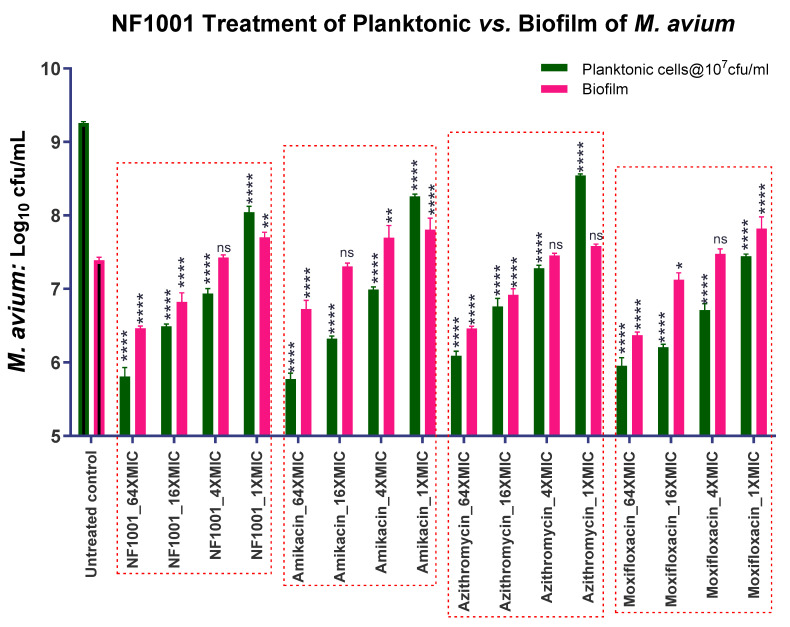
MIC/activity of NF1001 and SoC drugs against Mav biofilm. Matured biofilm of Mav exposed to compound/drugs for seven days at 37 °C. Data represent the mean ± SD of three biological replicates. Statistical significance was evaluated by one-way ANOVA (Dunnett’s test) compared to the untreated (bacteria only) control (**** *p* < 0.0001, *** *p* < 0.001, ** *p* < 0.01, * *p* < 0.1, or, ns = non-significant). The graph represents the residual log_10_ cfu/mL of Mav. Green filled bars represent the residual log_10_ cfu/mL of planktonic populations of Mav, while the filled pink bars show residual log_10_ cfu/mL of biofilm populations compared to the respective untreated controls. The untreated control bars represent the respective growth of the planktonic and the biofilm populations.

The combination MIC/activity of NF1001 with SoC drugs against NTM biofilms:

- The MIC and killing potential of NF1001 against planktonic and biofilm forms of Mabs (cfu reduction): NF1001 in the 96-well format at 64× MIC (64 µg/mL) was able to inhibit and kill Mabs cells in the biofilm to reduce the cfu by 0.63 log_10_/_mL_, on par with AZI (0.54), one of the reference drugs used in the treatment of NTM (Figure 6). However, NF1001 demonstrated a moderate killing of Mabs in the biofilm compared to the other reference drugs used. The reference drug AMI demonstrated a better kill (Emax = 1.39). The rank order of the Emax (log_10_ cfu reduction) was AMI (1.39) > MOX (1.09) > NF1001 (0.63) > AZI (0.54).
- The MIC/Activity of NF1001 against the biofilm of Mav: NF1001 showed an Emax of 0.91 log_10_ cfu reduction in the Mav biofilm at the highest concentration tested at 64× MIC (64 µg/mL) in a 96-well format. AMI showed a similar response (Emax = 0.91) by NF1001 against the Mav biofilm (Figure 7). The rank order of the Emax_64µg/mL_ (log_10_ cfu reduction at 64 µg/mL) was NF1001 (0.91) = AMI (0.91) > MOX (0.87) > AZI (0.65). The activity of NF1001 was on par with AMI and was better than MOX and AZI, the reference drugs tested against Mav (Table 4).

**Figure 8:**
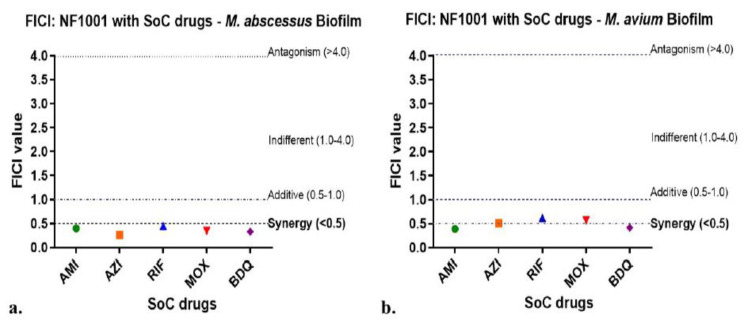
NF1001 in combination with SoC drugs against biofilms of Mabs and Mav. The FICI values were plotted in the graphs of Mabs (**a**) and Mav (**b**) for NF1001 in combination with different SoC drugs as these values appear in different zones of drug-drug interaction outcomes, e.g., synergy, additive, indifferent or antagonism. Symbols are uniform across respective combination drugs for both the cultures (Mav, and Mabs): filled circles in green = AMI, filled squares in orange = AZI, filled upright triangles in blue= RIF, filled inverted triangles in red= MOX, filled diamonds in purple= BDQ. FICI values for Mabs (**a**) and Mav (**b**), respectively, in the range of 0.26–0.45 and 0.39–0.62. No antagonism was observed (FICI < 4.0), indicating no interaction.

The result of combination MIC assays against NTM biofilms. The FICI of NF1001 combinations with five SoC drugs (AMI, AZI, RIF, MOX, BDQ) against Mabs and Mav biofilms were plotted (Figure 7):

NF1001 demonstrated synergism or it was additive with all the SoC drugs tested against the biofilms of both NTM species, Mabs and Mav. NF1001 did not show antagonism with any of the SoC drugs against these NTM (FICI < 4.0). No antagonism was observed (FICI < 4.0) with any of the five SoC drugs tested (Figure 8a,b). None of the combinations had FICI > 1.0, suggesting that NF1001 has a great treatment potential in combination. The data used to calculate the FICI are in the Supplementary Figures S2 and S3.

## 4. Discussion

Biofilms are a robust physiological state of pathogenic microbes including NTM and represent NTM’s major survival strategy in the hostile environment. In the present study, we evaluated the activity of the lead anti-NTM compound NF1001 against the planktonic phase versus the biofilms of the two medically important NTM, Mabs and Mav. NF1001 is effective not only against planktonic NTM cells but also reduces the NTM cell burden in the biofilm.

The lead compound NF1001 has potent pan-NTM activity against the planktonic stage of various NTM species studied including Mav/MAC and Mabs (MIC range 0.06 to 2 µg/mL). NF1001 demonstrated a clear concentration-dependent anti-NTM activity against Mabs and Mav as an important PK/PD parameter from the killing kinetics study. Preliminary PK studies suggest that NF1001 rapidly distributes from blood into tissues, and achieves high lung concentrations. These high lung concentrations may be relevant in treating pulmonary infections. However, further in vivo PK/PD studies are required to gain better insight into the dose–response relationship of NF1001 to understand its main PK/PD driver.

NTM treatment includes a combination of poorly tolerated oral/intravenous antimycobacterial drug regimens for a prolonged time period. Mabs has developed an intrinsic/acquired resistance to many existing antibiotics, particularly to macrolides, the cornerstone of NTM SoC treatment options [14,31], thus making it extremely difficult or ineffective. The NF1001 compound demonstrated synergy or was additive with anti-NTM SoC drugs against planktonic or even biofilm populations of both Mabs and Mav (FICI range 0.1 to 1.0). There was no antagonism observed for any of the SoC drugs or the new drugs (MOX and BDQ) tested. Therefore, a novel alternative treatment option like NF1001 with the best fit in SoC combination regimens can fill in the gap for better treatment options.

Additionally, NF1001 exhibited potent activity (0.06 to 2 µg/mL) against all tested drug-resistant or -sensitive clinical strains of Mabs and Mav/MAC, including MDR strains. Out of a total of 31 clinical isolates tested, 10 were AMI-R (Mabs = 5 and MAC = 5 (Mav = 2, Mint = 3)), 9 isolates were resistant to CLA (Mabs = 7, Mav = 2), and 6 strains were resistant to both CLA and AMI (Mabs = 4 and MAC = 2 (Mav = 1, Mint = 1)) that inhibit protein synthesis machinery. Most of the peptides or thiopeptides like NF1001 exert their antibacterial activity via the inhibition of ribosomal protein synthesis [45,46]. The potent activity of NF1001 against clinical isolates of NTM resistant to the protein synthesis inhibitors (AMI and CLA) clearly indicates a novel mechanism distinct from AMI and CLA. However, an in-depth target identification and a mode-of-action analysis of NF1001 need to be established.

NF1001 is non-toxic in nature. It did not demonstrate any toxicity to THP-1, HepG2, and A549 cell lines, indicating a safe compound to be progressed further. It penetrates well into the intracellular milieu in THP-1 macrophages. Predominantly intracellular, an ideal treatment option for NTM-PD would be activity against the biofilm as well as intracellular infections. NF1001 exhibited potent activity against intracellular Mav and Mabs. AMI is one of the important drugs for the treatment of NTM infections. However, AMI has limited activity against intracellular NTM because of its poor penetration and accumulation in macrophages, unless it has liposomal encapsulation [47].

Biofilms and planktonic cell counterparts are different entities in terms of physiology, growth rates, and their ability to resist stress conditions (host immune system and anti-mycobacterial agents). Mycobacterial biofilms are proven to be more resistant to conventional antibiotics than planktonic bacteria due to complex genetic and phenotypic differences, limited antibiotic penetration, and high lipid content and hydrophobicity of the biofilm matrix [5,17,18,19]. We developed biofilms of NTM (Mabs and Mav) amenable to high throughput screening and successfully demonstrated the anti-biofilm potential of NF1001, a novel anti-NTM compound compared to three SoC drugs, AMI, AZI, and MOX. However, regardless of a good in vitro anti-planktonic profile, NF1001 demonstrated moderate activity against Mabs’ biofilm. The rank order of NF1001 vs. the other drugs tested for the in vitro activity of the individual antibiotics examined against planktonic and biofilm forms of *M. abscessus* and MAC in terms of reduction in CFU indicates that NF1001 may be progressed further, since it is equipotent against drug-resistant populations as well:

(i) Planktonic *M. abscessus* (amikacin ~moxifloxacin > NF1001 > azithromycin)
(ii) Biofilm *M. abscessus* (amikacin ~moxifloxacin > NF1001 > azithromycin).
(iii) Planktonic MAC (moxifloxacin > NF1001 ~amikacin > azithromycin)
(iv) Biofilm MAC (NF1001 ~amikacin ~azithromycin ~moxifloxacin)

The present study reiterates that Mabs biofilms are much harder to kill—consistent with earlier reports implicating the development of phenotypic antibiotic tolerance through aggregation, lower metabolism, and metabolic dormancy [48,49].

In general, the biofilm matrix restricts the penetration of antibiotics to reach underlying heterogeneous bacterial populations. But, many drugs like Fluoroquinolones, Rifamycins, and Ampicillin efficiently cross these matrices and show efficacy [50]. It is high time to discover and develop novel anti-NTM compounds that penetrate into biofilms and act by novel mechanisms, in order to work on drug-resistant/drug-tolerant NTM and exhibit synergy with the existing treatment options. NF1001 meets most of these criteria, being potent against planktonic NTM and biofilm forms of Mav and Mabs, the causative agents of opportunistic infections in CF patients [51,52,53,54,55]. We report here the following: (1) NF1001 is a novel, first-in-class anti-biofilm compound which can inhibit and reduce the microbial load in NTM biofilms alone or in combination with SoC anti-NTM drugs. (2) There was no antagonism observed with any of the five SoC drugs tested against the planktonic or the biofilm populations of NTM pathogens tested. It considerably reduced the bacterial load of biofilm populations. (3) Our studies suggest that NF1001 is non-cytotoxic, exhibited potent activity against intracellular Mav and Mabs, has a unique mechanism of action, and kills microbial populations in biofilms. (4) The unique feature of NF1001 is that it is a pan-mycobacteria active compound, potent against AMI/CLA-sensitive as well as multi-drug (AMI/CLA)-resistant NTM clinical isolates that are resistant to classical protein synthesis inhibitors (AMI, AZI). Potent against Mav and Mabs biofilm populations, NF1001 is currently being evaluated for its activity in mouse models of relevant NTM infections. In summary, this study suggests that NF1001 can reduce bacterial burden in biofilm, and it holds a significant potential to be developed as a new combination regimen for the treatment of drug-resistant NTM infections and NTM-PD, more importantly, for CF patients.

## Figures and Tables

**Table 1:** Summary of MIC and MBC of NF1001 and reference drugs against various NTM **(**µg/mL).

NTMs	*M. abscessus* ATCC 19977		*M. avium* ATCC 19698		*M. gordonae* ATCC 14470		*M. intracellulare* ATCC 13950		*M. kansasii* ATCC 12478		*M. nonchromogenicum* ATCC 19530	
Compounds	MIC	MBC	MIC	MBC	MIC	MBC	MIC	MBC	MIC	MBC	MIC	MBC
NF1001	0.5	2	1	1	0.5	2	0.06	1	1	4	0.5	2
Moxifloxacin	1	2	1	2	2	4	0.5	1	2	8	2	16
Rifampicin	8	16	0.25	1	0.25	8	0.06	0.25	0.125	16	0.125	32
Clarithromycin	0.5	4	0.5	2	0.5	1	0.125	0.5	0.5	2	0.25	4
Amikacin	2	4	1	4	2	8	1	2	4	16	2	8

**Table 2:** Cytotoxicity of test and reference compounds against various mammalian cells and intracellular efficacy (IE) of those in THP-1 macrophages infected with Mabs and Mav, respectively.

Compounds	Highest Conc. Tested (µg/mL)	Cytotoxic Concentration (µg/mL) vs. Different Mammalian Cells			Intracellular Efficacy (IE) of Compounds in THP-1 Macrophages (IE E_max_)	
					*M. abscessus* ATCC 19977	*M. avium* ATCC 19698
		HepG2	A549	THP-1	IE D-1 E_max_	IE D-7 E_max_
NF1001	64	>64	>64	>64	0.82	1.13
MOX	64	>64	>64	>64	1.07	1.46
RIF	64	>64	>64	>64	ND	1.19
CLA	64	>64	>64	>64	0.87	1.30
AZI	64	>64	>64	>64	0.69	ND
AMI	64	>64	>64	>64	ND	ND
Menadione	32	8	4	8	ND	ND

Only the best three effective drugs in comparison to NF1001 were tested for intracellular activity against the two NTM tested (*M. abscessus*: AZI, CLA, and MOX; *M. avium*: RIF, CLA, and MOX), and were non-cytotoxic (>64 µg/mL). Menadione was used as a cytotoxicity-positive drug control, showed cytotoxicity (4–8 µg/mL). Abbreviation ND = Not Done.

**Table 3:** MIC of NF1001 against clinical isolates of NTM: Mabs, Mav and Mint strains.

MIC of NF1001 in Planktonic NTM		MIC Range (µg/mL)		
SN	*M. abscessus* (*n* = 20)	MIC (Test)	MIC (QC)	
		NF1001	Amikacin	Clarithromycin
1	*M. abscessus abscessus* ATCC 19977	1	2	0.5
2	*M. abscessus abscessus* Clin#1	1	2	0.5
3	*M. abscessus abscessus* Clin#2	0.5	>64	>64
4	*M. abscessus abscessus* Clin#3	0.125	>64	>64
5	*M. abscessus abscessus* Clin#4	0.5–1	64	>64
6	*M. abscessus abscessus* Clin#5	0.25	>64	>64
7	*M. abscessus abscessus* Clin#6	0.125–0.25	>64	0.5
8	*M. abscessus abscessus* Clin#7	0.25–0.5	>64	2
9	*M. abscessus abscessus* Clin#8	0.5–1	>64	2
10	*M. abscessus abscessus* Clin#9	0.125–0.25	>64	1
11	*M. abscessus abscessus* Clin#10	0.25	4	>64
12	*M. abscessus abscessus* Clin#11	0.125–0.25	4	64
13	*M. abscessus abscessus* Clin#12	0.5–1	4	16
14	*M. abscessus abscessus* Clin#13	0.125–0.25	4	16
15	*M. abscessus abscessus* Clin#14	0.125	4	16
16	*M. abscessus abscessus* Clin#15	0.25	4	>64
17	*M. abscessus massiliense* Clin#1	1 to 2	2	>64
18	*M. abscessus massiliense* Clin#2	0.25–0.5	1	0.125–0.25
19	*M. abscessus massiliense* Clin#3	1	8	0.5–1
20	*M. abscessus massiliense* Clin#4	0.25–0.5	64	0.5–1
SN	*M. avium*/MAC (*n* = 14)	NF1001	Amikacin	Clarithromycin
1	*M. avium paratuberculosis* ATCC 19698	0.25–0.5	1–2	0.5
2	*M. avium* Clin#1	0.5–1	8	8
3	*M. avium* Clin#2	2	16	0.5–1
4	*M. avium* Clin#3	1	16	1
5	*M. avium* Clin#4	1	2	64
6	*M. avium* Clin#5	0.5	1–2	>64
7	*M. avium* Clin#6	2	2	1
8	*M. avium* Clin#7	2	1	0.5
9	*M. intracellulare* ATCC 13950	0.06–0.125	1	0.25
10	*M. intracellulare* Clin#1	0.5	16	16
11	*M. intracellulare* Clin#2	0.5	16	0.5–1
12	*M. intracellulare* Clin#3	0.5–1	16	0.5–1
13	*M. intracellulare* Clin#4	0.5	1	1
14	*M. intracellulare* Clin#5	1	16	0.5

NF1001 has pan-NTM potency across thirty-four different reference (ATCC) and clinical NTM isolates, irrespective of their drug-resistance profiles (drug-S or drug-R). Clinical isolates were procured from Dr. Charles Daley, NJH, Co US, and the WT NTM from ATCC.

**Table 4:** MIC of NF1001 and SoC and other drugs on biofilms of Mabs and Mav.

MIC of NF1001 and SoC against Biofilm		
Drugs	MIC (µg/mL)	
	*M. abscessus* ATCC 19977	*M. avium* ATCC 19698
NF1001	4	2
Amikacin (AMI)	16	8
Azithromycin (AZI)	16	32
Rifampicin (RIF)	64	16
Moxifloxacin (MOX)	4	8
Bedaquiline (BDQ)	2	2

SoC drugs: AMI, AZI, and RIF. Other drugs: MOX and BDQ.

## Data Availability

The data from the present study can be requested from the corresponding author.

## Supplementary Materials

The following supporting information can be downloaded at: https://www.mdpi.com/article/10.3390/pathogens13010040/s1. Figure S1: Representative biofilm picture of *M. abscessus* in a 12-well and 96-well format; Figure S2: Combination MIC of NF1001 and SoC against biofilm of *M. abscessus*; Figure S3: Combination MIC of NF1001 and SoC against biofilm of *M. avium*.
