# Supplementary material for: A Novel Inhibitor against the Biofilms of Non-Tuberculous Mycobacteria
Source: Pathogens. 2023 Dec 31;13(1):40. doi: 10.3390/pathogens13010040 (PMC10819454; doi:10.3390/pathogens13010040)
Supplement: Supplementary file 1 [file pathogens-13-00040-s001.zip › Figure S3.pdf]

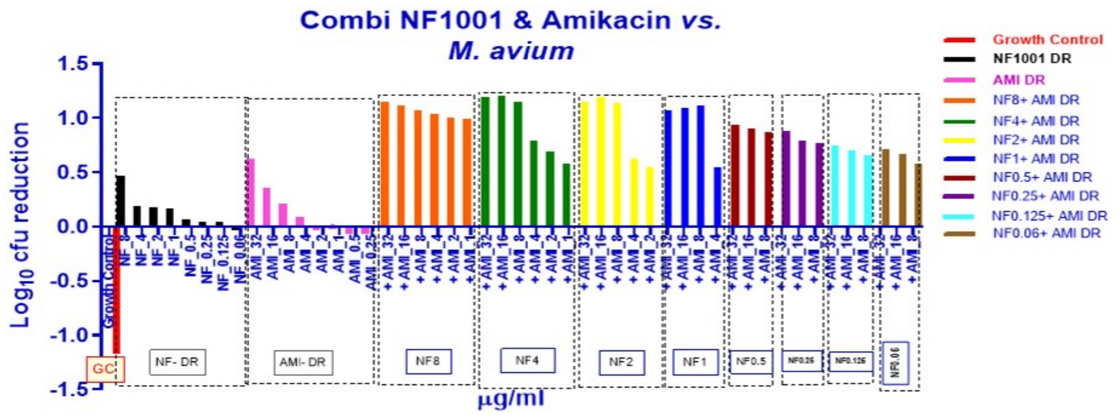
Combi NF1001 & Amikacin vs.
M. avium
Log10 cfu reduction
µg/ml
GC
NF- DR
AMI- DR
NF8
NF4
NF2
NF1
NF0.5
NF0.25
NF0.125
NF0.06
Growth Control
NF1001 DR
AMI DR
NF8+ AMI DR
NF4+ AMI DR
NF2+ AMI DR
NF1+ AMI DR
NF0.5+ AMI DR
NF0.25+ AMI DR
NF0.125+ AMI DR
NF0.06+ AMI DR

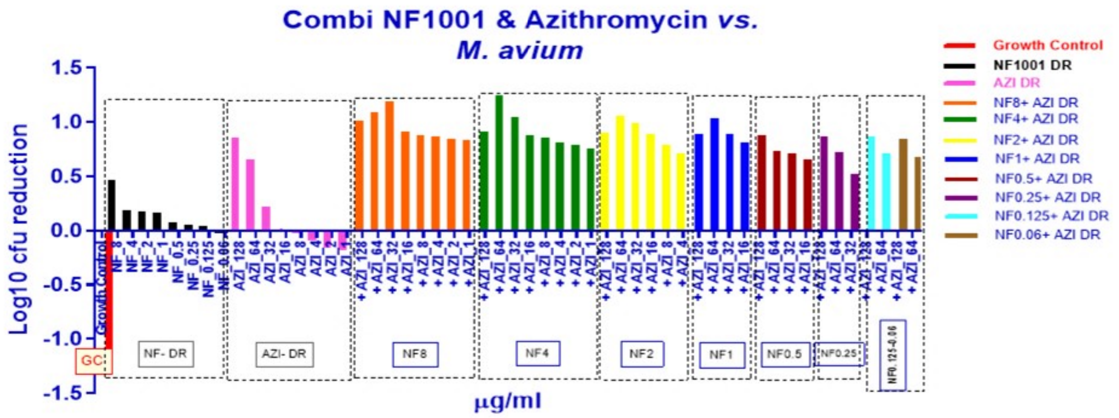
Combi NF1001 & Azithromycin vs.
M. avium
Log10 cfu reduction
µg/ml
GC
NF- DR
AZI- DR
NF8
NF4
NF2
NF1
NF0.5
NF0.25
NF0.125-0.06
Growth Control
NF1001 DR
AZI DR
NF8+ AZI DR
NF4+ AZI DR
NF2+ AZI DR
NF1+ AZI DR
NF0.5+ AZI DR
NF0.25+ AZI DR
NF0.125+ AZI DR
NF0.06+ AZI DR

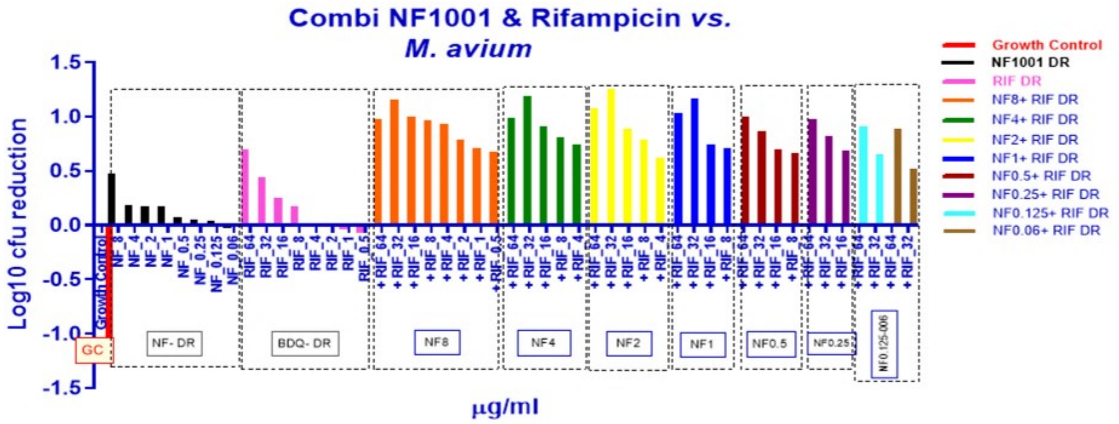
Combi NF1001 & Rifampicin vs.
M. avium
Log10 cfu reduction
µg/ml
GC
NF- DR
BDQ- DR
NF8
NF4
NF2
NF1
NF0.5
NF0.25
NF0.125-006
Growth Control
NF1001 DR
RIF DR
NF8+ RIF DR
NF4+ RIF DR
NF2+ RIF DR
NF1+ RIF DR
NF0.5+ RIF DR
NF0.25+ RIF DR
NF0.125+ RIF DR
NF0.06+ RIF DR

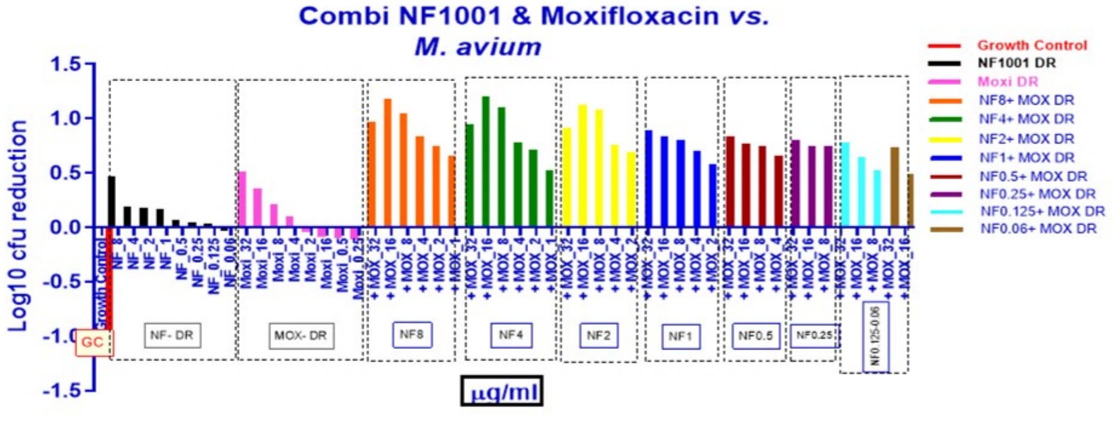
Combi NF1001 & Moxifloxacin vs.
M. avium
Log10 cfu reduction
µg/ml
GC
NF- DR
MOX- DR
NF8
NF4
NF2
NF1
NF0.5
NF0.25
NF0.125-0.06
Growth Control
NF1001 DR
Moxi DR
NF8+ MOX DR
NF4+ MOX DR
NF2+ MOX DR
NF1+ MOX DR
NF0.5+ MOX DR
NF0.25+ MOX DR
NF0.125+ MOX DR
NF0.06+ MOX DR

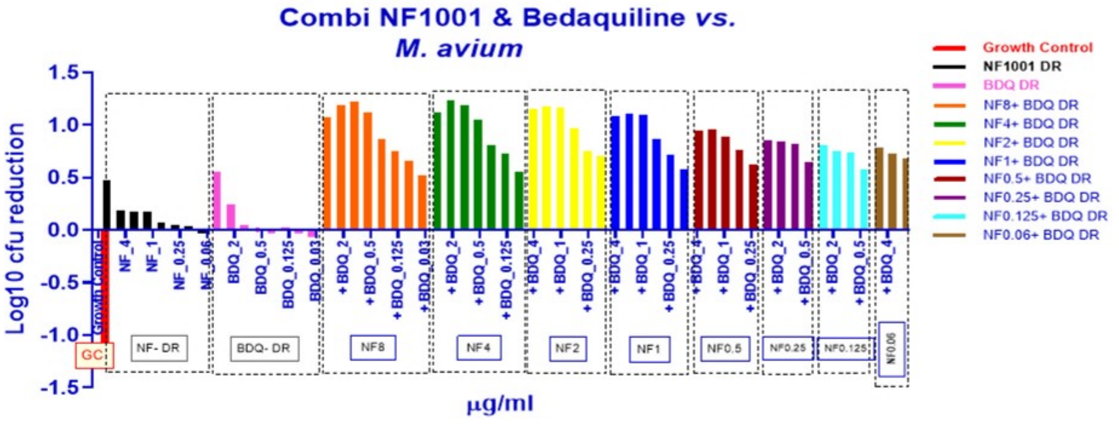
Combi NF1001 & Bedaquiline vs.
M. avium
Log10 cfu reduction
µg/ml
GC
NF- DR
BDQ- DR
NF8
NF4
NF2
NF1
NF0.5
NF0.25
NF0.125
NF0.06
Growth Control
NF1001 DR
BDQ DR
NF8+ BDQ DR
NF4+ BDQ DR
NF2+ BDQ DR
NF1+ BDQ DR
NF0.5+ BDQ DR
NF0.25+ BDQ DR
NF0.125+ BDQ DR
NF0.06+ BDQ DR
