# Supplementary figures and images for: A Novel Inhibitor against the Biofilms of Non-Tuberculous Mycobacteria
Source: Pathogens. 2023 Dec 31;13(1):40. doi: 10.3390/pathogens13010040 (PMC10819454; doi:10.3390/pathogens13010040)

a.

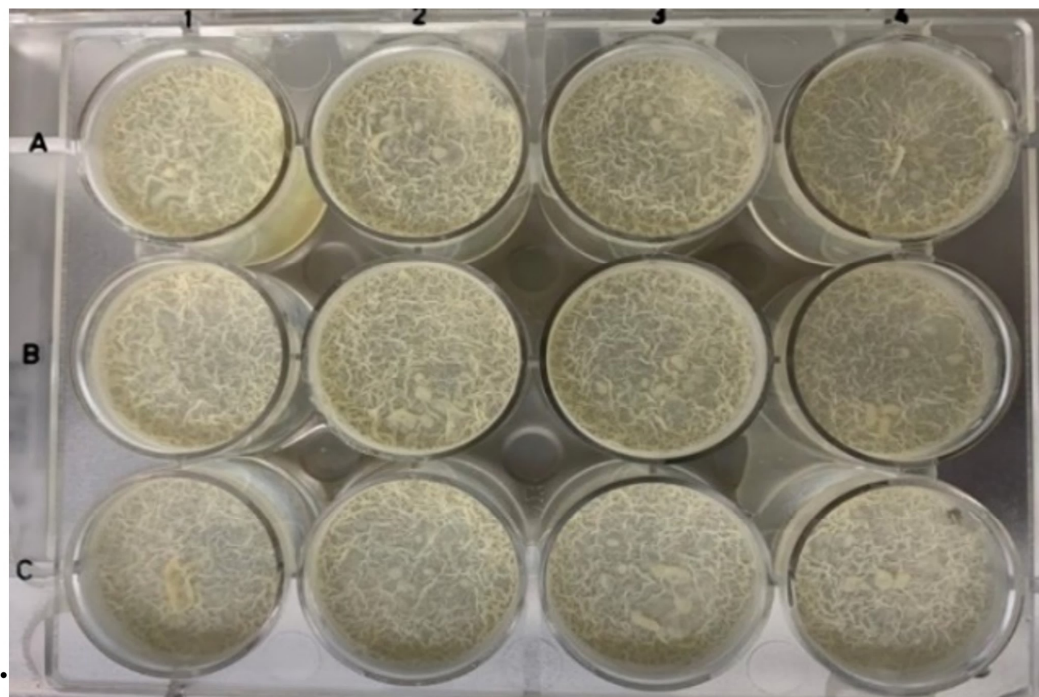

b.

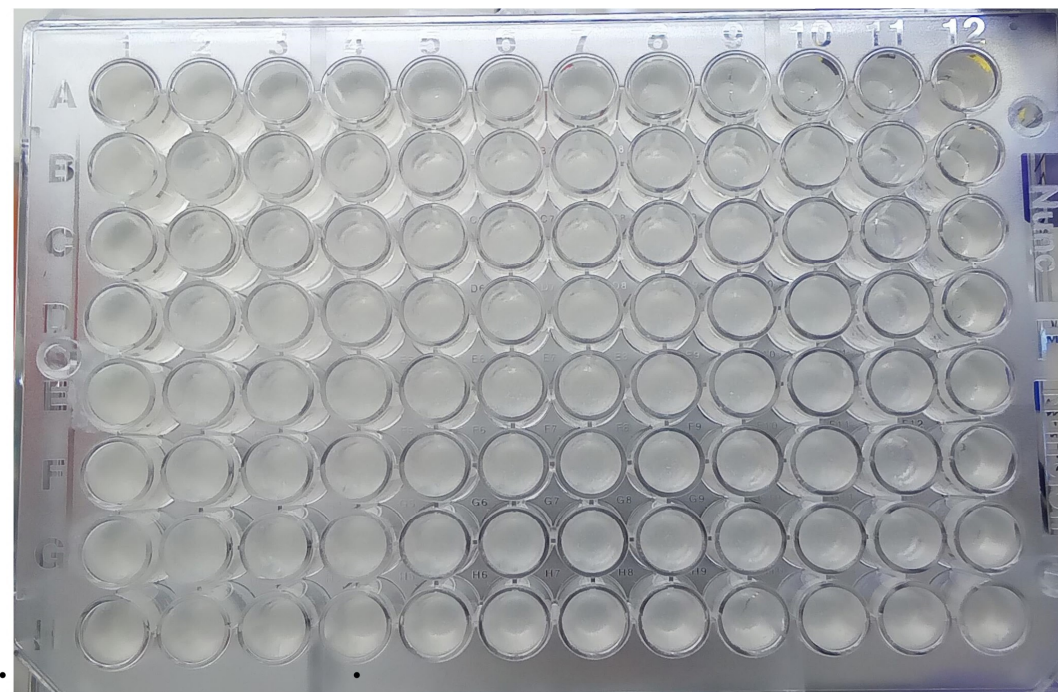

Supplement: Supplementary file 1 [file pathogens-13-00040-s001.zip › Figure S1.pdf]
